# Igf1R/InsR function is required for axon extension and corpus callosum formation

**DOI:** 10.1371/journal.pone.0219362

**Published:** 2019-07-18

**Authors:** Jing Jin, Priyadarshini Ravindran, Danila Di Meo, Andreas W. Püschel

**Affiliations:** 1 Institut für Molekulare Zellbiologie, University of Münster, Münster, Germany; 2 Cells-in-Motion Cluster of Excellence, University of Münster, Münster, Germany; Osaka University, JAPAN

## Abstract

One of the earliest steps during the development of the nervous system is the establishment of neuronal polarity and the formation of an axon. The intrinsic mechanisms that promote axon formation have been extensively analyzed. However, much less is known about the extrinsic signals that initiate axon formation. One of the candidates for these signals is Insulin-like growth factor 1 (Igf1) that acts through the Igf1 (Igf1R) and insulin receptors (InsR). Since Igf1R and InsR may act redundantly we analyzed conditional cortex-specific knockout mice that are deficient for both *Igf1r* and *Insr* to determine if they regulate the development of the cortex and the formation of axons *in vivo*. Our results show that Igf1R/InsR function is required for the normal development of the embryonic hippocampus and cingulate cortex while the lateral cortex does not show apparent defects in the *Igf1r*;*Insr* knockout. In the cingulate cortex, the number of intermediate progenitors and deep layer neurons is reduced and the corpus callosum is absent at E17. However, cortical organization and axon formation are not impaired in knockout embryos. In culture, cortical and hippocampal neurons from *Igf1r*;*Insr* knockout embryos extend an axon but the length of this axon is severely reduced. Our results indicate that Igf1R/InsR function is required for brain development in a region-specific manner and promotes axon growth but is not essential for neuronal polarization and migration in the developing brain.

## Introduction

Mature neurons are highly polarized cells with distinct axonal and somatodendritic compartments. The development of their complex morphology depends on multiple pathways that are regulated by extrinsic signals [1,2]. During the development of the mammalian brain, neurons are generated in the ventricular and subventricular zone (VZ and SVZ) by neural progenitor cells [2,3]. Subsequently, the newborn neurons become polarized and extend an axon and a leading process before initiating their radial migration into the cortical plate (CP) [1,4,5]. This differentiation step is called the multi-to-bipolar transition. Neurons isolated from the embryonic cortex or hippocampus undergo a similar polarization in culture with the extension of a single axon and several dendrites [2,5,6,7,8,9,10]. Manipulation of signaling pathways in cultured neurons allowed the identification of numerous factors that are required for neuronal polarization and axon formation.

One of the extracellular signals that promote axon extension in culture is Igf1 [2,11,12]. The growth factors Igf1, Igf2 and insulin are recognized by both the Igf1 receptor (Igf1R, encoded by the *Igf1r* gene in mice) and the insulin receptors (InsR, *Insr*) [13,14,15,16]. Igf1R and InsR can also form functional heterodimers that have similar affinities for Igf1 and insulin [17,18,19]. All three ligands are expressed in the developing nervous system and are important for neurogenesis and neuronal differentiation [17,20,21]. *Igf1r* knockout mice display severe growth defects and die after birth [13,22]. Biochemical studies and the analysis of knockouts for *Igf1r*, *Insr* and their ligands revealed that Igf1 and Igf2 can signal through both Igf1R and InsR [13,23,24]. Inactivation of *Igf1r* specifically in the nervous system using different *Nestin-Cre* lines results in a severe reduction of brain size [15,21,25,26]. Knockout of *Igf1* or *Igf2* also reduces brain size, due to the reduced proliferation of progenitors and a defect in myelination [15,21,27,28,29]. The proliferation of neural progenitors is stimulated by Igf2 from the cerebrospinal fluid [21]. Interfering with IgfR1 signaling reduces the proliferation of neural progenitors and the number of oligodendrocytes. In addition, a specific subset of microglia is an important source of Igf1 and required for primary myelination [27].

Igf1R was also implicated in the establishment of neuronal polarity in cultures of hippocampal neurons upstream of phosphatidylinositol-4,5-bisphosphate 3-kinase [12,30,31,32,33]. Igf1 stimulates the expansion of the plasma membrane by the insertion of specialized vesicles in the growth cone to promote axon extension [34,35,36]. Addition of a function-blocking antibody or knockdown of Igf1R interferes with the formation of axons but it remains to be shown that Igf1R is required for axon formation also *in vivo* [12,30,31,32,33]. The knockout of Igf1R or InsR alone does not impair the organization of cortical layers in the mouse brain [21,37]. However, a knockdown of Igf1R by in utero electroporation of the cortex blocks the transition from a multi- to a bipolar morphology and the migration of cortical neurons, which accumulate mainly in the VZ/SVZ [11].

Here we investigate the *in viv*o function of Igf1R/InsR signaling for cortical development by analyzing cortex-specific, conditional *Igf1R*;*Insr* knockout mice. Our results reveal that *Igf1r*;*Insr* double mutants exhibit region-specific deficits in cortical development. A thinning of the cingulate cortex, an agenesis the corpus callosum (CC) and a severe reduction of the hippocampus were observed at E17 while no defects were detectable in the lateral cortex. Axon formation is not impaired in the cortex *in vivo* but axon length is severely reduced in cultured neurons. Our analysis indicates that Igf1R/InsR signaling impacts progenitor cells in a region-dependent manner during neuronal development but is not essential for the formation of axons *in vivo*.

## Results

### Loss of hippocampus but normal cortical architecture in *Igf1r*;*Insr* knockout embryos

**Fig 1:**
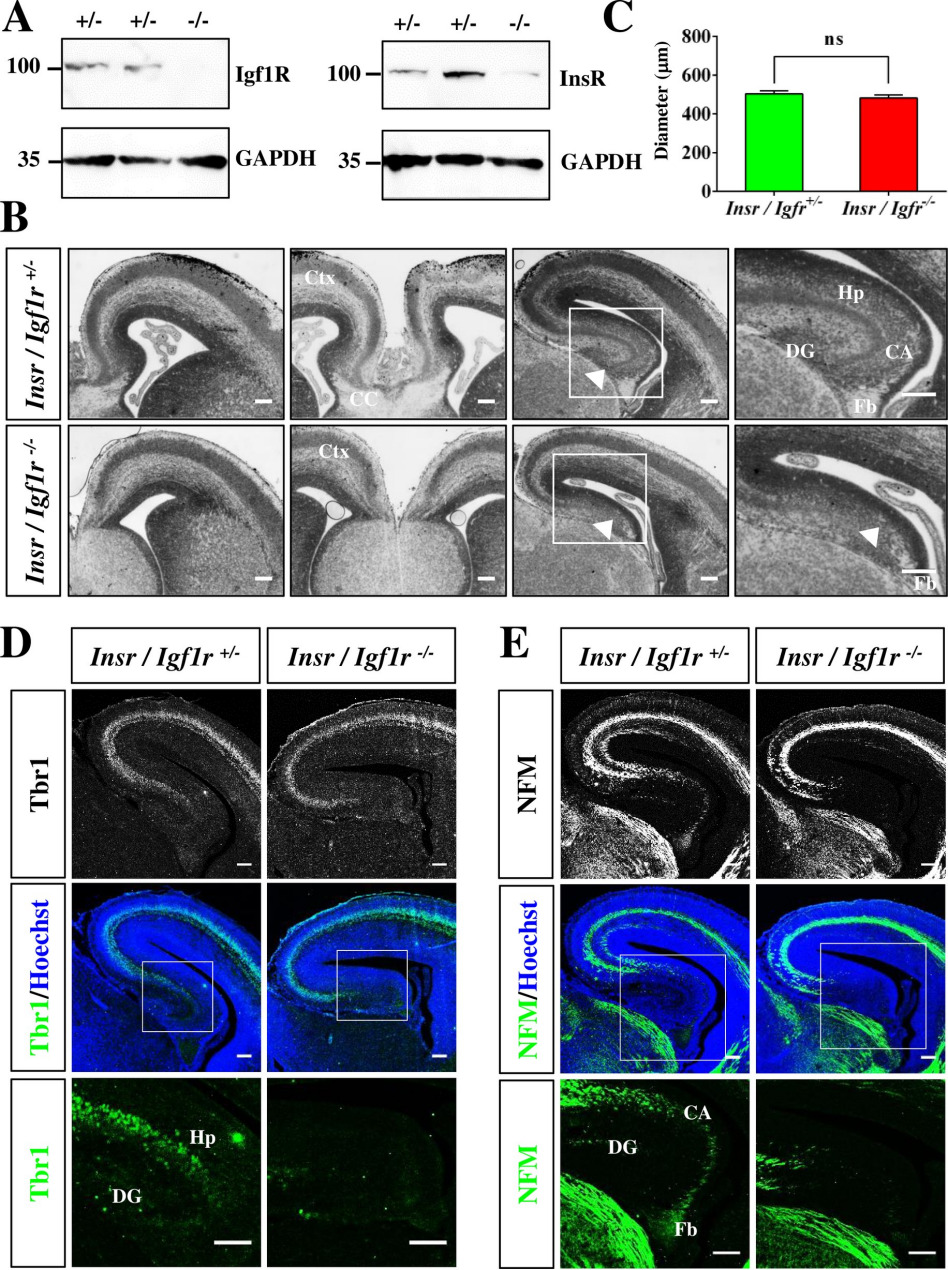
Igf1r/Insr-Emx1 KO mice show defects in the corpus callosum and hippocampus. (A) The expression of InsR and Igf1R in the cortex of heterozygous (+/-, *Igf1r*/*Insr*^+/-^: *Insr*^flox/+^;*Igf1r*^flox/+^;*Emx1-Cre*^C/+^) and homozygous (-/-, *Igf1r*/*Insr*^-/-^: *Insr*^flox/flox^;*Igf1r*^flox/flox^;*Emx1-Cre*^C/+^) E17 knockout embryos was analyzed by Western blot. Residual signals result from non-neural cells that are not affected by the cortex-specific knockout mediated by *Emx1-Cre*. The loading of comparable amounts of protein was verified using an anti-GAPDH antibody. The molecular weight is indicated in kDa. (B) Coronal sections from the brain of heterozygous (*Ifg1r*/*Insr*^+/-^) or homozygous (*Igf1r*/*Insr*^-/-^) E17 Igf1r/Insr-Emx1 KO embryos were stained with hematoxylin and eosin. A higher magnification of the regions marked by a white frame is shown in the right panels. Arrowheads mark the region of the hippocampus that is missing in Igf1r/Insr-Emx1 KO embryos. The scale bars are 100 μm (left panel) and 20 μm (right panel), respectively. (C) The thickness of the lateral cerebral cortex was determined at the level of the corpus callosum from the midpoint of the ventricular surface perpendicular to the pial surface in sections from heterozygous or homozygous Igf1r/Insr-Emx1 KO mice (n = 3 brains; means ± s.e.m.; ns, not significant (p>0.05); Mann-Whitney U-test). (D, E) Sections from the hippocampus of heterozygous (*Ifg1r*/*Insr*^+/-^) and homozygous (*Igf1r*/*Insr*^-/-^) E17 knockout embryos were stained with Hoechst 33342 (blue), an anti-Tbr1 (D, green) and an anti-NFM (E, green) antibody. The lower panel in (D, E) shows a higher magnification of the region marked by a white frame. The scale bar is 100 μm (upper panel) and 20 μm (lower panel), respectively. CA: Cornu Ammonis; Ctx: cortex; CC: corpus callosum; Hp: hippocampus; NE: neuroepithelium; DG: dentate gyrus; Fb: fimbria.

To investigate the role of Igf1R/InsR signaling for neuronal development *in vivo* we generated cortex-specific knockout mice deficient for both Igf1R and InsR by crossing the conditional *Igf1r*^flox/flox^ and *Insr*^flox/flox^ lines with the *Emx1-Cre* line that mediates a cortex-specific knockout beginning at E10.5 (Igf1R/IR-Emx1 KO: *Igf1r*^flox/flox^;*Insr*^flox/flox^;*Emx1-Cre*^C/+^) [38,39,40,41,42,43]. Western blot analysis confirmed that the expression of Igf1R and InsR was almost completely undetectable in the cortex of Igf1r/Insr-Emx1 KO E17 embryos (Fig 1A).

A histological analysis did not reveal major defects in the organization of the neocortex in Igf1R/IR-Emx1 KO mutants at E17 (Fig 1B). There was no significant difference in the diameter of the lateral cortex at the level of the CC when comparing heterozygous (504 ± 15 μm) and homozygous Igf1r/Ir-Emx1 KO embryos (482 ± 16 μm) (Fig 1C) in contrast to previously reported complete *Igf1r* and conditional *Igf1r*;*Nestin-Cre* knockout mice [21]. However, the hippocampus (cornu ammonis and dentate gyrus) was missing in all Igf1r/Insr-Emx1 KO embryos analyzed (n = 6; Fig 1B). This phenotype is more severe than that reported previously for *Igf1r*;*Nestin-Cre* knockout mice [26]. The phenotype was confirmed by staining with an anti-Tbr1 antibody as a marker for post-mitotic neurons (Fig 1D). Corresponding to the reduced number of neurons, staining with a neurofilament medium chain (NFM) antibody as axonal marker revealed a loss of axons in the Igf1r/Insr-Emx1 knockout hippocampus (Fig 1E).

### *Igf1r*/*Insr* function is required in the cingulate but not lateral cortex

**Fig 2:**
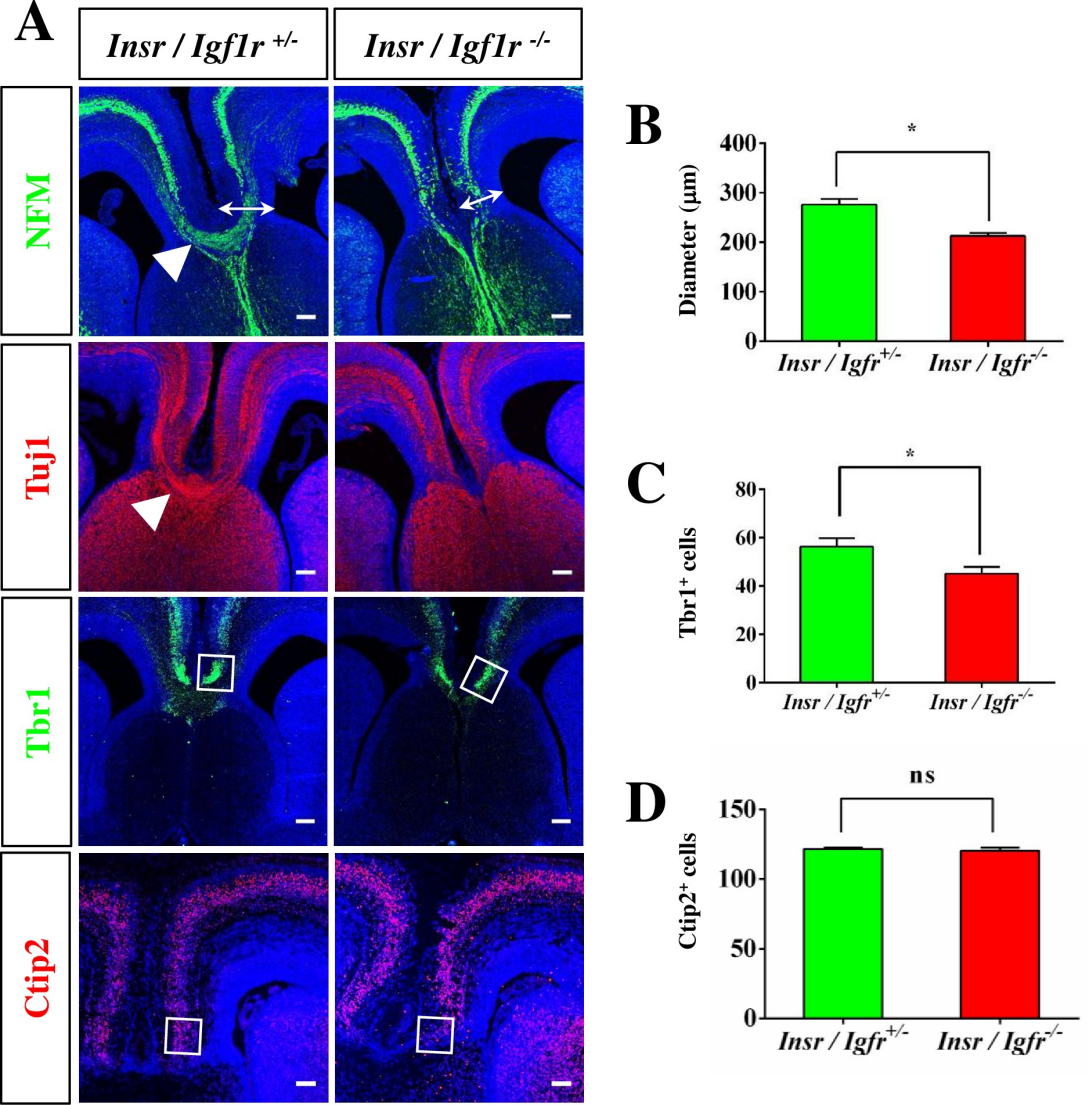
Agenesis of the corpus callosum in Igf1r/Insr-Emx1 KO embryos. (A-D) Coronal sections from the cortex of heterozygous (*Ifg1r*/*Insr*^+/-^) and homozygous (*Igf1r*/*Insr*^-/-^) E17 knockout embryos were stained with Hoechst 33342 (blue) and an anti-NFM (green), the Tuj1 (red), an anti-Tbr1 (green) or an anti-Ctip2 (red) antibody as indicated. The scale bar is 100 μm. Arrowheads mark the corpus callosum (heterozygous controls). (B) The radial diameter of the cingulate cortex was determined in sections from heterozygous or homozygous Igf1r/Insr-Emx1 KO mice (arrow in A; n = 3 brains; means ± s.e.m.; *, p<0.05; ns, not significant (p>0.05); compared to heterozygous controls; Mann-Whitney U-test). (C, D) The number of Tbr1- (C) or -Ctip2-positive cells (D) in a column of 100 μm width from the cingulate cortex of heterozygous and homozygous Igf1r/Insr-Emx1 KO embryos is shown (area analyzed is marked in A; n = 3 brains; means ± s.e.m; *, p<0.05 compared to heterozygous controls; Mann-Whitney U-test).

In addition to the defect in hippocampal development a thinning of the cingulate cortex and a loss of the CC was evident in the E17 Igf1r/Insr-Emx1 KO brain (Fig 1B). The diameter of the cingulate cortex (Fig 2A and 2B; Igf1r/Insr-Emx1 KO: 213 ± 4 μm, heterozygous control: 276 ± 7 μm) and the number of Tbr1^+^ neurons in this region was reduced (Fig 2A and 2C, S1 Fig; KO: 47 ± 3 cells per 100 μm, control: 60 ± 4 cells per 100 μm). Staining for the deep and upper layer markers Ctip2 and Satb2 did not reveal a significant difference in the cingulate cortex (Fig 2A and 2D, S3 and S4 Figs; KO: 147 ± 3 cells per 100 μm, control: 146 ± 2 cells per 100 μm). The markers NFM and Tuj1 showed that axons fail to cross the midline to form the CC and remain largely ipsilaterally (Fig 2A).

**Fig 3:**
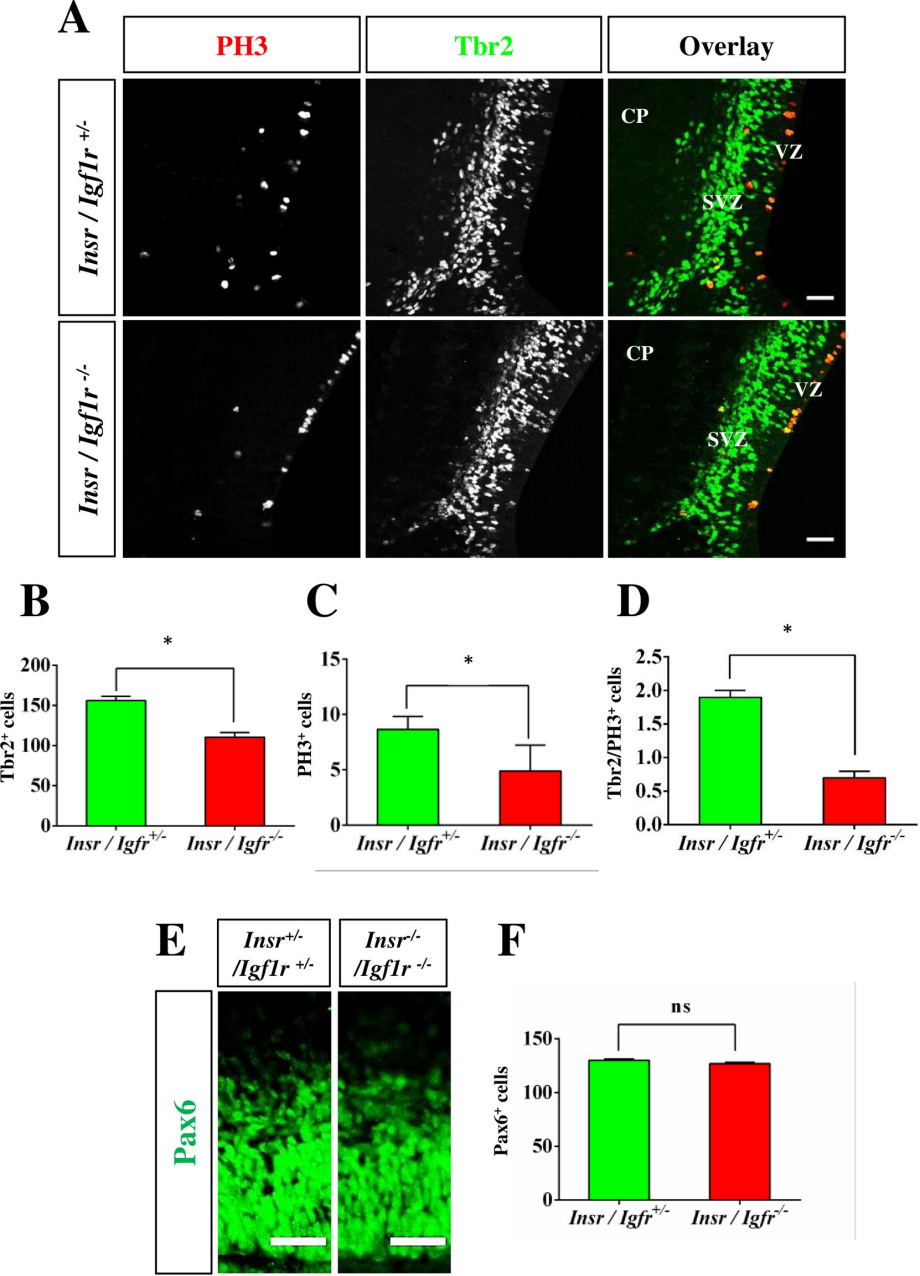
Reduced number of intermediate progenitor cells in the Igf1r/Insr- Emx1 KO cingulate cortex. (A) Coronal sections from the cortex of heterozygous (*Ifg1r*/*Insr*^+/-^) and homozygous (*Igf1r*/*Insr*^-/-^) E17 knockout embryos were stained with anti-PH3 (red) and anti-Tbr2 antibodies (green). The scale bar is 100 μm. (B—D) The number of cells positive for Tbr2 (B), PH3 (C) or for both Tbr2 and PH3 (D) in a column of 100 μm width was quantified in the cingulate cortex of heterozygous and homozygous Igf1r/Insr-Emx1 KO embryos (n = 3 brains; means ± s.e.m.; *, p<0.05 compared to heterozygous controls; Mann-Whitney U-test. CP: cortical plate; SVZ: subventricular zone; VZ: ventricular zone. (E) Coronal sections from the cortex of heterozygous (*Ifg1r*/*Insr*^+/-^) and homozygous (*Igf1r*/*Insr*^-/-^) E17 knockout embryos were stained with an anti-Pax6 antibody (green). The scale bar is 50 μm. (F) The number of cells positive for Pax6 in a column of 100 μm width was quantified in the cingulate cortex (n = 3 brains; means ± s.e.m.; ns, p>0.05; Student’s t-test).

Igf1R and InsR regulate the proliferation of neuronal stem cells and the number of progenitor cells is reduced in *Igf1r*;*Nestin-Cre* knockout mice [21,29,43]. Inactivation of Tbr2 that is expressed in intermediate progenitors (IPCs) results in a failure of axons to cross the midline and the agenesis of the CC [44,45]. To investigate if intermediate progenitors (IPCs) are affected in the Igf1r/Insr-Emx1 KO we stained for Tbr2 and phospho-Histone 3 (PH3) as markers for IPCs and mitotic cells, respectively (Fig 3A–3D). The number of Tbr2^+^ progenitor cells was significantly reduced in the cingulate cortex (Fig 3B; KO mice: 111 ± 6 cells per 100 μm; control: 156 ± 6 cells per 100 μm). Consistent with the reduced number of Tbr2^+^ progenitor cells, the number of PH3^+^ mitotic cells (Fig 3C; I KO: 5 ± 2 cells per 100 μm, control: 9 ± 1 cells per 100 μm) and the number of IPCs that are positive for Tbr2 and PH3 were reduced (KO: 0.7 ± 0.1 cells per 100 μm, control: 1.9 ± 0.1 cells per 100 μm; Fig 3D). By contrast, the number of Pax6^+^ apical progenitors was not different compared to control (Fig 3E and 3F, S5 Fig; KO mice: 127 ± 2 cells per 100 μm; control: 130 ± 2 cells per 100 μm).

**Fig 4:**
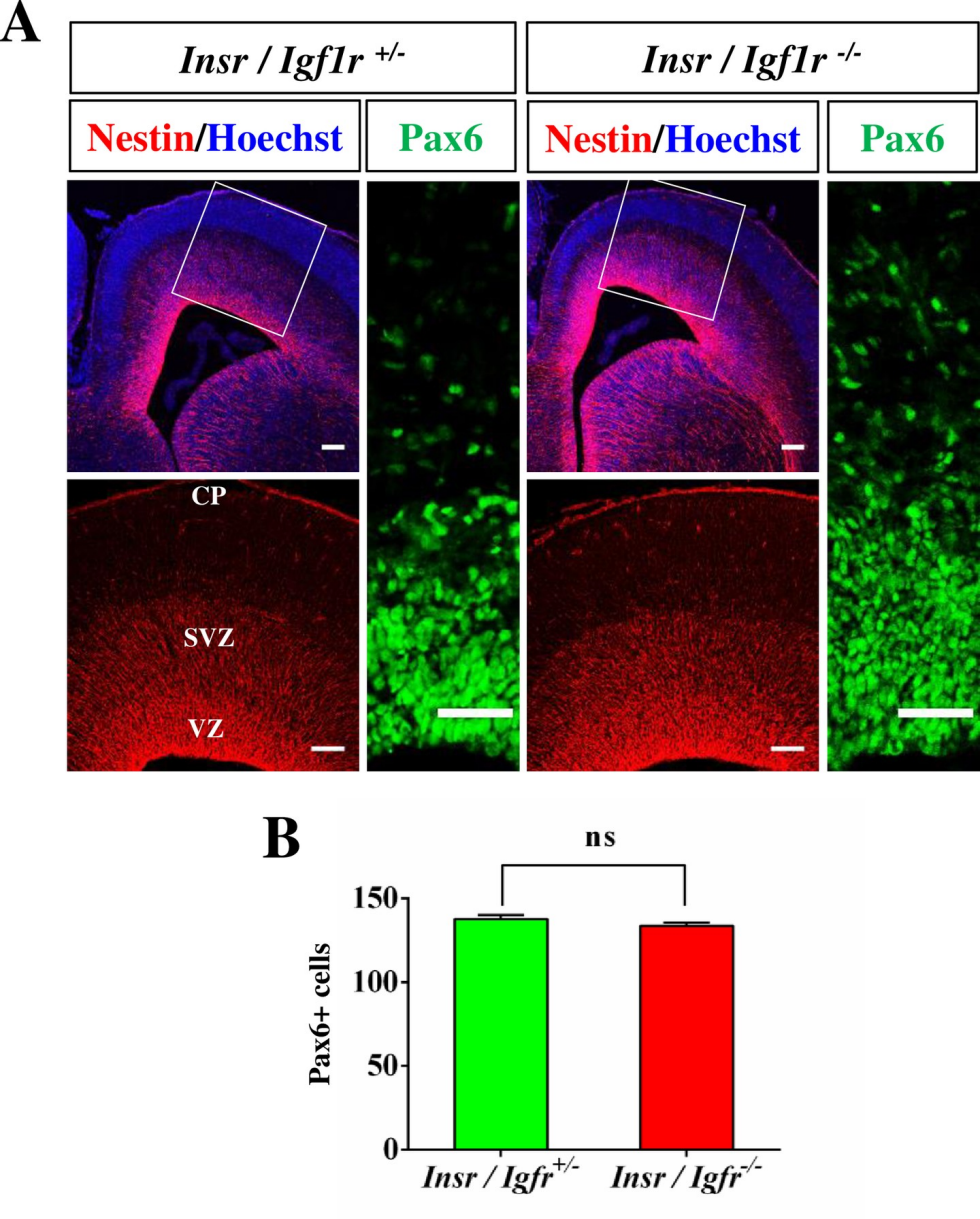
Radial glia cells are not affected in the lateral cortex of the Igf1r/Insr-Emx1 KO. (A) Coronal sections from the cortex of heterozygous (*Ifg1r*/*Insr*^+/-^) and homozygous (*Igf1r*/*Insr*^-/-^) E17 knockout embryos were stained with anti-nestin (red) or anti-Pax6 antibodies (green) and Hoechst 33342 (blue). A higher magnification of the region marked in (A) is shown in the lower panel. The scale bar is 100 μm (left panels) and 50 μm (right panel), respectively. (B) The number of cells positive for Pax6 in a column of 100 μm width was quantified in the lateral cortex (n = 3 brains; means ± s.e.m.; ns, p>0.05; Student’s t-test).

**Fig 5:**
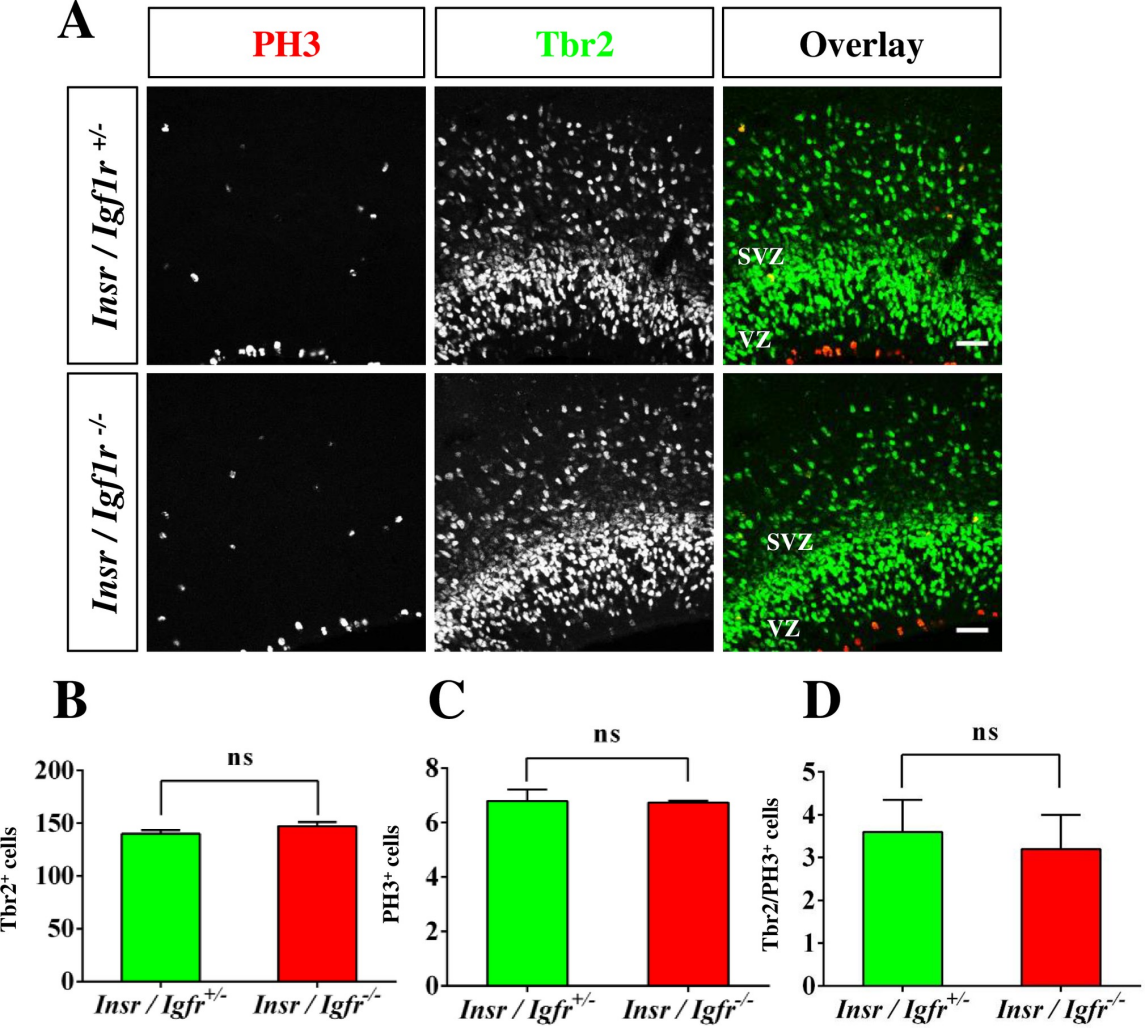
Progenitor cells are not affected in the lateral cortex of the Igf1r/Insr-Emx1 KO. (A) Coronal sections from the lateral cortex of heterozygous (*Ifg1r*/*Insr*^+/-^) and homozygous (*Igf1r*/*Insr*^-/-^) knockout E17 embryos were stained with anti-PH3 (red) and anti-Tbr2 antibodies (green). The scale bar is 20 μm. (B—D) The number of cells positive for Tbr2 (C), PH3 (D) or both Tbr2 and PH3 (E) in a column of 100 μm width was quantified in the lateral cortex of Igf1r/Insr-Emx1 KO and heterozygous embryos (n = 3 brains; means ± s.e.m; ns, p>0.05; Student’s t-test). SVZ: subventricular zone; VZ: ventricular zone.

In contrast to the cingulate cortex, no defect was observed in the lateral cortex of Igf1r/Insr-Emx1 KO embryos (Fig 1B). Staining with anti-nestin and anti-Pax6 antibodies, which mark radial glia cells (RGCs; Fig 4A and 4B, S5 Fig), and an anti-Tbr2 antibody also did not reveal defects in the lateral cortex (Fig 5A and 5B, S6 Fig). The numbers of Tbr2^+^ IPCs (Fig 5B; KO: 148 ± 4 cells per 100 μm; heterozygous control: 140 ± 4 cells per 100 μm) and mitotic PH3^+^ cells (Fig 5C; KO: 7 ± 1 cells per 100 μm; control: 7 ± 1 cells per 100 μm) were similar in the cortex of Igf1r/Insr-Emx1 KO mice and controls. The number of mitotic IPCs that are positive for both of Tbr2 and PH3 was not significantly changed (Fig 5D; KO: 3.2 ± 0.8 cells per 100 μm, control: 3.6 ± 0.7 cells per 100 μm). These results indicate that the loss of Igf1R/InsR signaling affects IPCs in the cingulate but not the lateral cortex, which may cause the agenesis of the CC similar to the phenotype of the Tbr2 knockout.

### Igf1R/InsR function is required for axon extension but not neuronal polarity

**Fig 6:**
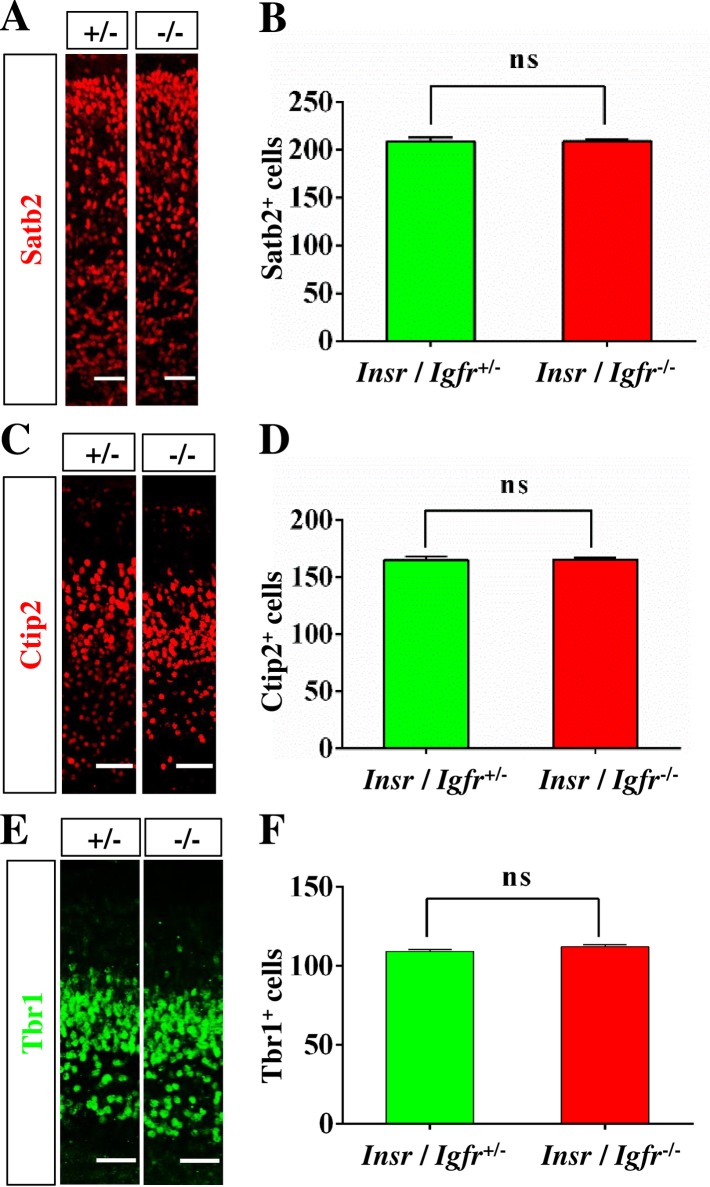
The development of cortical layers is not affected in the Igf1r/Insr-Emx1 KO cortex. (A, C, E) Coronal sections from the lateral cortex of heterozygous (+/-: *Ifg1r*/*Insr*^+/-^) and homozygous (-/-: *Igf1r*/*Insr*^-/-^) E17 knockout embryos were stained with anti-Satb2 (red), Ctip2 (red) or -Tbr1 antibody (green). The scale bar is 50 μm. (B, D, F) The number of Satb2-, Ctip2- or Tbr1-positive cells in a column of 100 μm width from the cortex of heterozygous and homozygous from Igf1r/Insr-Emx1 KO embryos is shown (n = 3 brains; means ± s.e.m; ns, p>0.05 compared to heterozygous controls; Student’s t-test).

A knockdown of Igf1R was reported to arrest neurons in the multipolar state and block their migration into the cortical plate [11]. To investigate defects in the formation of the cortical layers, we stained sections from the brain of E17 embryos with anti-Tbr1, -Ctip2 and -Satb2 antibodies as markers for deep and upper layer neurons (Fig 6 and S1–S3 Figs). Neither the position nor the number of Tbr1^+^, Ctip2^+^ or Satb2^+^ neurons was significantly different in Igf1r/Insr-Emx1 KO mice compared to heterozygous controls (Fig 6B, 6D and 6F). These results indicate that inactivation of *Igf1r* and *Insr* does not interfere with the formation of cortical layers.

**Fig 7:**
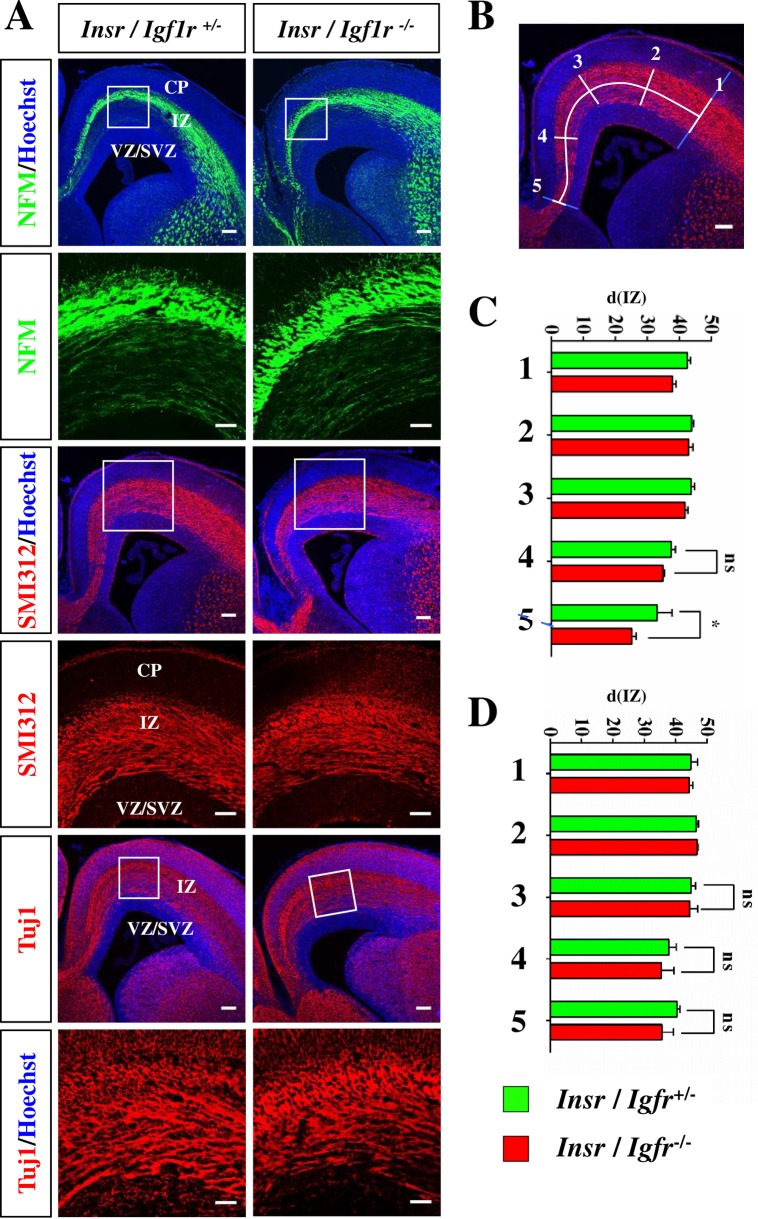
Igf1R/InsR signaling is not essential for axon formation in the embryonic cortex. (A) Coronal sections from the cortex of heterozygous (*Ifg1r*/*Insr*^+/-^) and homozygous (*Igf1r*/*Insr*^-/-^) E17 knockout embryos were stained with an anti-NFM (axons, green), the SIM312 (axons, red), the Tuj1 antibody (neurons, red) and Hoechst 33342 (blue). A higher magnification of the marked region is shown in the lower panels. The scale bar is 100 μm (upper panels) and 20 μm (lower panels), respectively. CP: cortical plate; IZ: intermediate zone; VZ/SVZ: ventricular zone/subventricular zone. (B-D) The relative diameter (d(IZ)) of the IZ (white line) and the cortical wall (gray line) was determined at five positions. To select comparable positions a line from the corticoseptal (5) to the corticostriatal boundary (1) along the middle of the IZ was subdivided into four sections of equal length. The diameter was determined at the indicated positions 1–5 and the relative diameter of the IZ calculated as d(IZ) = (d_iz_/d_c_)*100. (C, D) Coronal sections were stained with an anti-NFM (C) or the SIM312 antibody (D) and the relative diameter of the IZ determined at the five positions indicated (n = 3 brains; means ± s.e.m.; ns, p>0.05; *, p<0.05 compared to heterozygous controls; Mann-Whitney U-test).

It has been reported that Igf1R is required for the polarization of hippocampal neurons in culture [12]. However staining with an anti-NFM antibody as axonal marker did not reveal defects in axon formation in the intermediate zone (IZ) of the Igf1r/Insr-Emx1 KO at E17 (Fig 7A and S7 Fig). Staining with the SMI-312 or Tuj1 antibody detecting neuron-specific class III beta-tubulin also confirmed the formation of axons (Fig 7A). A quantification of the diameter of the IZ did not show a significant difference between heterozygous controls and the Igf1r/Insr-Emx1 KO except for the corticoseptal boundary, which displays a small reduction in diameter due to the defects in the CC (Fig 7B–7D). Thus, Igf1R/InsR function is not essential for the formation of axons in the developing embryonic cortex until E17.

**Fig 8:**
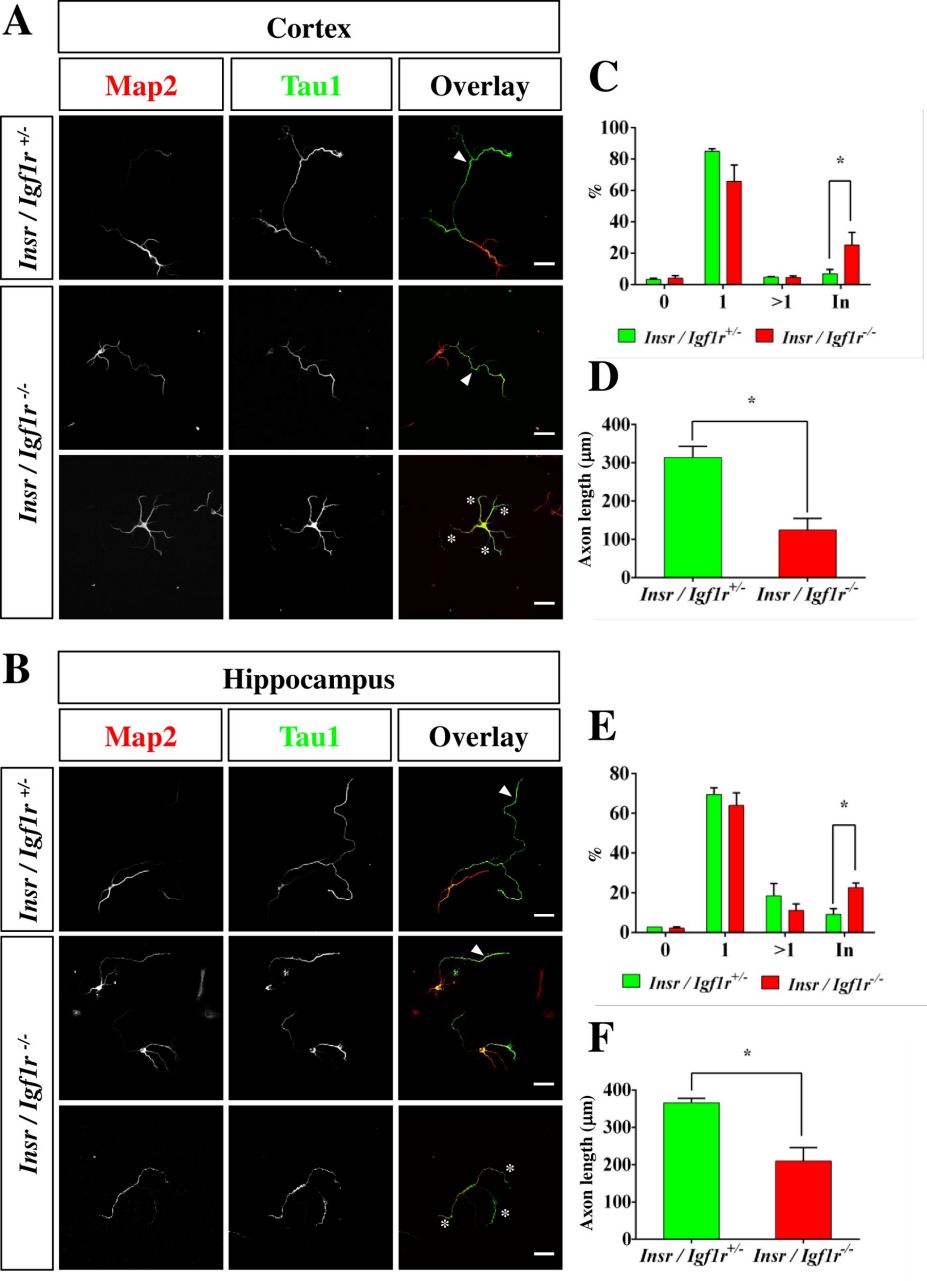
The loss of Igf1R and InsR impairs axon extension by cultured cortical and hippocampal neurons. (A, B) Neurons from the cortex (A) or hippocampus (B) of heterozygous (*Ifg1r*/*Insr*^+/-^) and homozygous (*Igf1r*/*Insr*^-/-^) E17 knockout embryos were analyzed at 3 days in culture (d.i.v.) by staining with an anti-Map2 (red) and the Tau-1 (blue) antibody. The scale bar is 20 μm. The majority of *Igf1r*/*Insr*^-/-^ neurons extend a single axon (arrowhead, upper panels) but some *Igf1r*/*Insr*^-/-^ neurons extend neurites that are positive for both of Map2 and Tau-1 (asterisks, lower panels). (C, E) The percentage (%) of unpolarized neurons without an axon (0), polarized neurons with a single axon (1), neurons with multiple axons (>1) and with multiple indeterminate neurites positive for both of Map2 and Tau-1 (In) from the cortex (C) and hippocampus (E) is shown (3 experiments, n>100 neurons, means ± s.e.m.; *, p<0.05 compared to heterozygous mice; two-way ANOVA). (D, F) The length of cortical (D) and hippocampal axons (F) from homozygous and heterozygous knockout embryos is shown (3 experiments, n>50 neurons, means ± s.e.m.; *, p<0.05 compared to heterozygous mice; Student’s t-test).

To analyze the polarization of cultured neurons cortical and hippocampal neurons were isolated from E17 Igf1r/Insr-Emx1 KO embryos and stained at 3 days in culture with an anti-Map2 antibody as a marker for dendrites and the Tau-1 antibody as axonal marker (Fig 8A and 8B). The majority of cortical neurons extended a single axon (Fig 8A and 8C; KO: 66 ± 8%; control: 85 ± 2%) but their length was significantly reduced in neurons from Igf1r/Insr-Emx1 KO embryos from 314 ± 21 μm in controls to 125 ± 21 μm (Fig 8D). In addition, an increased proportion of cortical neurons (KO: 25 ± 6%) extended multiple neurites that were positive for both Map2 and Tau-1 compared to 7 ± 2% of the neurons from heterozygous controls (Fig 8C). Cultured hippocampal neurons showed a similar result (Fig 8B). The length of the axons was reduced (Fig 7F; KO: 209 ± 25 μm; control: 366 ± 8 μm) and 23 ± 2% of the neurons extended multiple neurites positive for Tau-1 and Map2 staining compared to 9 ± 2% in the heterozygous mutant (Fig 8E). Thus, Igf1R/InsR function is not essential for the formation of axons in culture and *in vivo* but is required for their growth.

## Discussion

Our analysis of conditional *Igfr1*;*Insr;Emx1-Cre* knockout mice revealed developmental defects that were restricted to the hippocampus and the cingulate cortex at E17 and an impairment of axon extension in cultured neurons. In culture, the majority of Igf1r/Insr-Emx1 KO neurons extended a single axon that was significantly shorter than controls. These results are consistent with the observation that Igf1 stimulates the extension of layer 5 corticospinal motor neurons [46]. However, severe deficits in axon formation were not detectable in the embryonic cortex. While our analysis of the Igf1r/Insr-Emx1 KO does not exclude the possibility that axon extension is impaired in some neurons the majority of neurons does not require Igf1R/InsR signaling *in vivo* to establish neuronal polarity and extend a single axon in contrast to previous studies based on the knockdown of Igf1R in cultured neurons [12].

The multi-to-bipolar transition of newborn neurons involves two polarization events, the extension of an axon and the formation of a leading process, which happen before they begin their radial migration into the CP [1,4,5]. The suppression of Igf1R in cortical neurons by RNAi was reported to block neuronal polarization and migration and leads to an accumulation of multipolar neurons in the VZ/SVZ [11]. A defect in the multi-to-bipolar transition and radial migration with an arrest in the IZ usually results in a severe disorganization of cortical layers and the absence of axons [47,48,49,50]. Our results show that the formation of cortical layers was not disrupted in Igf1r/Insr-Emx1 KO mice at E17 (Figs 4 and 5), which is consistent with previous analyses of *Igfr1* knockouts [15,21]. Thus, Igf1R/InsR signaling does not appear to be essential for the radial migration of cortical neurons at least until E17. It is possible that the defects in neuronal migration observed after knockdown result from sequence-dependent off-target effects [51]. Taken together, these results indicate that Igf1R/InsR signaling is not essential for the establishment of neuronal polarity and neuronal migration in the developing cortex.

Previous analysis of *Igf1r*;*Nestin-Cre* knockouts reported a decrease in the size of the cortex and hypomyelination [15,20,21,28,52,53]. This phenotype can be attributed to defects in neural progenitor proliferation and in primary myeliniation. While the cingulate cortex was reduced in the Igf1r/Insr-Emx1 KO at E17, the size of the lateral cortex was not significantly affected in contrast to the hippocampus that was poorly developed as reported previously [26]. The distinct phenotypes of the different knockout lines could result from differences in the Cre expression pattern. The *Emx1-Cre* line mediates a cortex-specific knockout that is more restricted than the *Nestin-Cre* transgenic line [21,26]. The different *Nestin-Cre* lines used in previous studies are active not only in the neuroepithelium along the whole anterio-posterior axis but also in tissues outside the nervous system [42,54,55,56,57,58], which may explain the more severe phenotype of *Igf1r*;*Nestin-Cre* knockout lines [21,26].

In the cingulate cortex the number of progenitors and neurons was reduced and the CC failed to form. An agenesis of the corpus callosum was also described at a later developmental stage for mutants, in which Igf1R was specifically inactivated in oligodendrocytes [29]. The defect in the CC of these mutants was observed at postnatal stages and results from an abnormal development of oligodendrocytes and defects in myelination. The phenotype of the Igf1r/Insr-Emx1KO indicates that Igf1r/Insr signaling is required for the formation of the CC also during embryonic development. The CC is formed mainly by the axons of layer 2, 3 and 5 neurons that cross the midline at the corticoseptal boundary [59,60,61]. It is pioneered by axons from the cingulate cortex that require the glial sling, glial wedge and glia within the indusium griseum to cross the midline and form the CC [62,63,64,65,66,67,68]. In Tbr2 knockout mice, axons fail to cross the midline and form the CC [44,45]. The reduced number of progenitors in the Igf1r/Insr-Emx1 KO may cause the agenesis of the CC by affecting the midline cell populations that guide axons across the midline indicating that IgfR1/InsR signaling is required for CC formation.

Taken together, these results indicate that Igf1R/InsR signaling regulates neuronal development in a region-specific manner and is required for axon growth. However, Igf1R/InsR signaling is not essential for neuronal polarization and migration *in vivo* in the embryonic brain until E17.

## Materials and methods

### Mice

To generate a cortex-specific conditional knockout for *Igf1r* and *Insr* (Igf1r/Insr-Emx1 KO: *Igf1r*^flox/flox^;*Insr*^flox/flox^;*Emx1-Cre*^C/+^) we crossed *Igf1r*^flox/flox^ [39] and *Insr*^flox/flox^ [38] with *Emx1*^Cre/Cre^ mice (Guo et al., 2000). *Emx1*-*Cre* mice were obtained from The Jackson Laboratory (Bar Harbor, Maine). All strains were kept in a C57Bl/6 background. Genotyping was done by PCR using the following primers: *Igf1r*: 5’-TCCCTCAGGCTTCATCCGCAA-3’ and 5’-CTTCAGCTTTGCAGGTGCACG-3’, *Insr*: 5’-GATGTGCACCCCATGTCT G-3’ and 5’-CTGAATAGCTGAGACCACAG-3’, *Emx*1 wt: 5’-AAGGTGTGGTTC CAGAATCG-3’ and 5’-CTCTCCACCAGAAGGCTGAG-3’, *Emx1*-*Cre*: 5’-AATG ACTAGGGGAACAATCAAGA-3’ and 5’-TCCAGGTATGCTCAGAAAACG-3’. Mice were housed at four to five per cage with a 12 h light/dark cycle (lights on from 07:00 to 19:00 h) at constant temperature (23°C) with *ad libitum* access to food and water. Adult mice were euthanized by cervical dislocation. All animal protocols were approved by the Landesamt für Natur, Umwelt und Verbraucherschutz Nordrhein-Westfalen.

### Antibodies

The following antibodies were used for Western blot analysis: InsR (1:100, catalog number 611276, BD Biosciences), anti-Igf1R (1:1000, 9750, Cell Signaling), and anti-GAPDH (1:1000, 9484, Abcam). HRP-coupled anti-rabbit (1:3000, 115035033, Dianova) and anti-mouse (1:3000, 111035033, Dianova) were used as secondary antibodies. To analyze cultured hippocampal neurons, we used anti-Map2 (1:1000, M4402, Sigma) and the Tau-1 (1:200, MB3420, Chemicon) antibodies. For immunofluorescence staining of paraffin sections the following antibodies were used: anti-nestin (1:100, 611658, BD Bioscience), anti-NFM (1:1000, 7794, Abcam), anti-PH3 (1:10, 9701 or 1:1000, 53348, both Cell Signaling), anti-Tbr1 (1:320, 31940, Abcam), anti-Tbr2 (1:800, 23345, Abcam), Tuj1 (1:1000, MAB1195, R&D Systems), SMI-312 (1:1000, 24574, Abcam) and goat secondary antibodies labeled with AlexaFluor-350, -488 or -594 (1:1000, Molecular Probes). For immunofluorescence staining of frozen sections, anti-Pax6 (1:200, 901301, BioLegend), anti-Ctip2 (1:100, 18465, Abcam), anti-Satb2 (1:100, sc-81376, SantaCruz Biotechnology), anti-NF160 (1:800, 64300, Abcam) and goat secondary antibodies labelled with AlexaFluor-488 or -594 (1:800, Molecular Probes) were used. Nuclei were stained with Hoechst 33342 (1:10000, C2110, Molecular Probes).

### Western blot analysis

The cortex was dissected from the brains of embryonic day 17 (E17) embryos and lysed in ice-cold RIPA buffer (1% NP40, 1% sodium desoxycholate, 0.1% SDS, 50mM HEPES (pH 7.4), 150mM NaCl, 10% Glycerol, 1.5mM MgCl_2_) for 1 h at 4°C. The lysate was cleared by centrifugation at 13,000rpm for 20 min at 4°C. The expression of Igf1R and InsR was analyzed by Western blot using primary antibodies diluted in blocking buffer (5% BSA in Tris-buffered saline (pH 7.4), 0.1% Tween-20) at 4°C and the enhanced chemiluminescence detection system (Uptima, Interchim UP99619A).

### Culture of cortical and hippocampal neurons

The isolated cortex or hippocampus was dissected from E17 mouse embryos in ice-cold Hanks' balanced salt solution (HBSS) and dissociated in 100μl trypsin solution (0.25% Trypsin/EDTA, Thermo Fisher Scientific) for 8 min at 37°C. Cells were plated at 40,000 cells/well in 24-well plates containing cover-slips coated with Poly-L-ornithine (15μg/ml, Sigma, P-3655) and cultured in Neurobasal medium with supplements (600μl, 1:50 B-27; 1:100 L-Glutamine, Thermo Fisher Scientific) at 37°C and 5% CO_2_ for 3 days.

### Immunofluorescence staining of cultured neurons

Cortical and hippocampal neurons were fixed in 4% paraformaldehyde (PFA)/15% sucrose in phosphate-buffered saline (PBS, PH 7.4) for 20 min at room temperature and permeablized in 0.1% Triton X-100/0.1% sodium citrate/PBS for 3 min on ice. Cells were blocked for 1 h at RT with blocking buffer (10% normal goat serum in 1×PBS, Thermo Fisher Scientific) and incubated with the primary antibody diluted in blocking buffer overnight at 4°C and secondary antibody diluted in blocking buffer for 90 min at room temperature.

### Histology

For a histological analysis, sections were stained with hematoxylin and eosin using standard procedures. Brains from E17 embryos were fixed in Carnoy’s solution (70% ethanol, 20% chloroform, 10% acetic acid) overnight at 4°C, dehydrated in xylene and embedded in paraffin. Coronal 10μm sections were cut using a microtome (Leica). Sections were deparaffinized, rehydrated and stained with Mayer’s Hemalum for 3 min, followed by 1 min wash in tap water, incubated in 0.5% HCl in 70% ethanol for 10 sec and washed again in tap water for 10 min. Freshly filtered 0.05% Eosin G was used for staining (1 min) followed by dehydration.

### Immunofluorescence staining of sections

To prepare frozen sections the brain of E17 embryos was fixed in 4% paraformaldehyde overnight at 4°C, put through a sucrose gradient (10%, 20%, 30% sucrose) and frozen in Tissue freezing medium (Leica). 10 μm coronal sections were cut using a cryostat (Leica) at -20°C.

Antigen-retrieval was performed by boiling sections in 10 mM sodium citrate buffer, 0.05% Tween20 (pH 6.0) in a microwave for 10 min at 650 watts followed by 400 watts for 10 min. The sections were blocked with 1% normal goat serum in PBS, 0.3% Triton X-100 for 1 h and stained with primary antibody diluted in blocking buffer overnight at 4°C and secondary antibodies for 90 min at room temperature. Neuronal morphology was analyzed using a Zeiss Axiophot microscope equipped with a Visitron CCD camera, and the SPOT Advanced Imaging software. Sections were imaged using a Zeiss 700 confocal laser scanning microscope and single planes are displayed. ImageJ was used for counting and measurement.

### Statistical analysis

The number of cells positive for the analyzed markers was determined in a column of the cortex (including VZ, SVZ, IZ, CP and the MZ) with a width of 100 μm [69]. In cases when the number of neurons positive for a marker was small all cells in the field of view (width 332 μm) were counted and the number normalized to a column with a width of 100μm (Figs 3D and 5D). To quantify the diameter of the IZ in Fig 7 we determined the diameter of the cortex (d_c_) and the IZ (d_iz_) at five positions and calculated the relative diameter of the IZ as d(IZ) = (d_iz_/d_c_)*100. To select comparable positions in sections from different embryos a line from the corticostriatal to the corticoseptal boundary along the middle of the IZ was subdivided into four sections of equal length and the diameter determined at these positions as indicated in Fig 7B. Data were analyzed by Mann-Whitney U-test, two-way ANOVA or Student’s t-test as indicated in the figure legends (Prism 5, Version 5.00, GraphPad Software and Microsoft Excel 2007). Unless specified otherwise, all values are means ± s.e.m. from at least three independent experiments.

## Supporting information

S1 Fig Distribution of Tbr1^+^ neurons in the Igf1r/Insr- Emx1 KO cortex. Coronal sections from the cortex of heterozygous (*Ifg1r*/*Insr*^+/-^) and homozygous (*Igf1r*/*Insr*^-/-^) E17 knockout embryos were stained with an anti-Tbr1 antibody (green) and Hoechst 33342 (blue). Sections were selected for analysis every 60 to 80 μm in the rostral to caudal direction beginning with the appearance of the corpus callosum. The scale bar is 100 μm.
(TIF)

S2 Fig Distribution of Ctip2^+^ neurons in the Igf1r/Insr- Emx1 KO cortex. Coronal sections from the cortex of heterozygous (*Ifg1r*/*Insr*^+/-^) and homozygous (*Igf1r*/*Insr*^-/-^) E17 knockout embryos were stained with an anti-Ctip2 antibody (green) and Hoechst 33342 (blue). Sections were selected for analysis every 60 to 80 μm in the rostral to caudal direction beginning with the appearance of the corpus callosum. The scale bar is 100 μm.
(TIF)

S3 Fig Distribution of Satb2^+^ neurons in the Igf1r/Insr- Emx1 KO cortex. Coronal sections from the cortex of heterozygous (*Ifg1r*/*Insr*^+/-^) and homozygous (*Igf1r*/*Insr*^-/-^) E17 knockout embryos were stained with an anti-Satb2 antibody (green) and Hoechst 33342 (blue). Sections were selected for analysis every 60 to 80 μm in the rostral to caudal direction beginning with the appearance of the corpus callosum. The scale bar is 100 μm.
(TIF)

S4 Fig The number of Ctip2 and Satb2^+^ neurons is not reduced in the cingulate cortex of the Igf1r/Insr- Emx1 KO cortex. (A, B) Coronal sections from the cortex of heterozygous (+/-: *Ifg1r*/*Insr*^+/-^) and homozygous (-/-: *Igf1r*/*Insr*^-/-^) E17 knockout embryos were stained with an anti-Ctip2 (A, red) or -Satb2 antibody (B, red). The scale bar is 100 μm. (B) The number of cells positive for Satb2 in a column of 100 μm width was quantified in the cingulate cortex of heterozygous and homozygous Igf1r/Insr-Emx1 KO embryos (n = 3 brains; means ± s.e.m.; *, p<0.05; Mann-Whitney U-test).
(TIF)

S5 Fig Distribution of Pax6^+^ apical progenitors in the Igf1r/Insr- Emx1 KO cortex. Coronal sections from the cortex of heterozygous (*Ifg1r*/*Insr*^+/-^) and homozygous (*Igf1r*/*Insr*^-/-^) E17 knockout embryos were stained with an anti-Pax6 antibody (green) and Hoechst 33342 (blue). Sections were selected for analysis every 60 to 80 μm in the rostral to caudal direction beginning with the appearance of the corpus callosum. The scale bar is 100 μm.
(TIF)

S6 Fig Distribution of Tbr2^+^ intermediate progenitors in the Igf1r/Insr- Emx1 KO cortex. Coronal sections from the cortex of heterozygous (*Ifg1r*/*Insr*^+/-^) and homozygous (*Igf1r*/*Insr*^-/-^) E17 knockout embryos were stained with an anti-Tbr2 antibody (green) and Hoechst 33342 (blue). Sections were selected for analysis every 60 to 80 μm in the rostral to caudal direction beginning with the appearance of the corpus callosum. The scale bar is 100 μm.
(TIF)

S7 Fig Igf1R/InsR signaling is required for the formation of the corpus callosum but not essential for axon formation in the embryonic cortex. Coronal sections from the cortex of heterozygous (*Ifg1r*/*Insr*^+/-^) and homozygous (*Igf1r*/*Insr*^-/-^) E17 knockout embryos were stained with an anti-NFM (axons, green), antibody and Hoechst 33342 (blue). A higher magnification of the corpus callosum is shown in the right panels. Sections were selected for analysis every 60 to 80 μm in the rostral to caudal direction beginning with the appearance of the corpus callosum The scale bar is 100 μm.
(TIF)
