# Supplementary figures and images for: Igf1R/InsR function is required for axon extension and corpus callosum formation
Source: PLoS One. 2019 Jul 18;14(7):e0219362. doi: 10.1371/journal.pone.0219362 (PMC6638864; doi:10.1371/journal.pone.0219362)

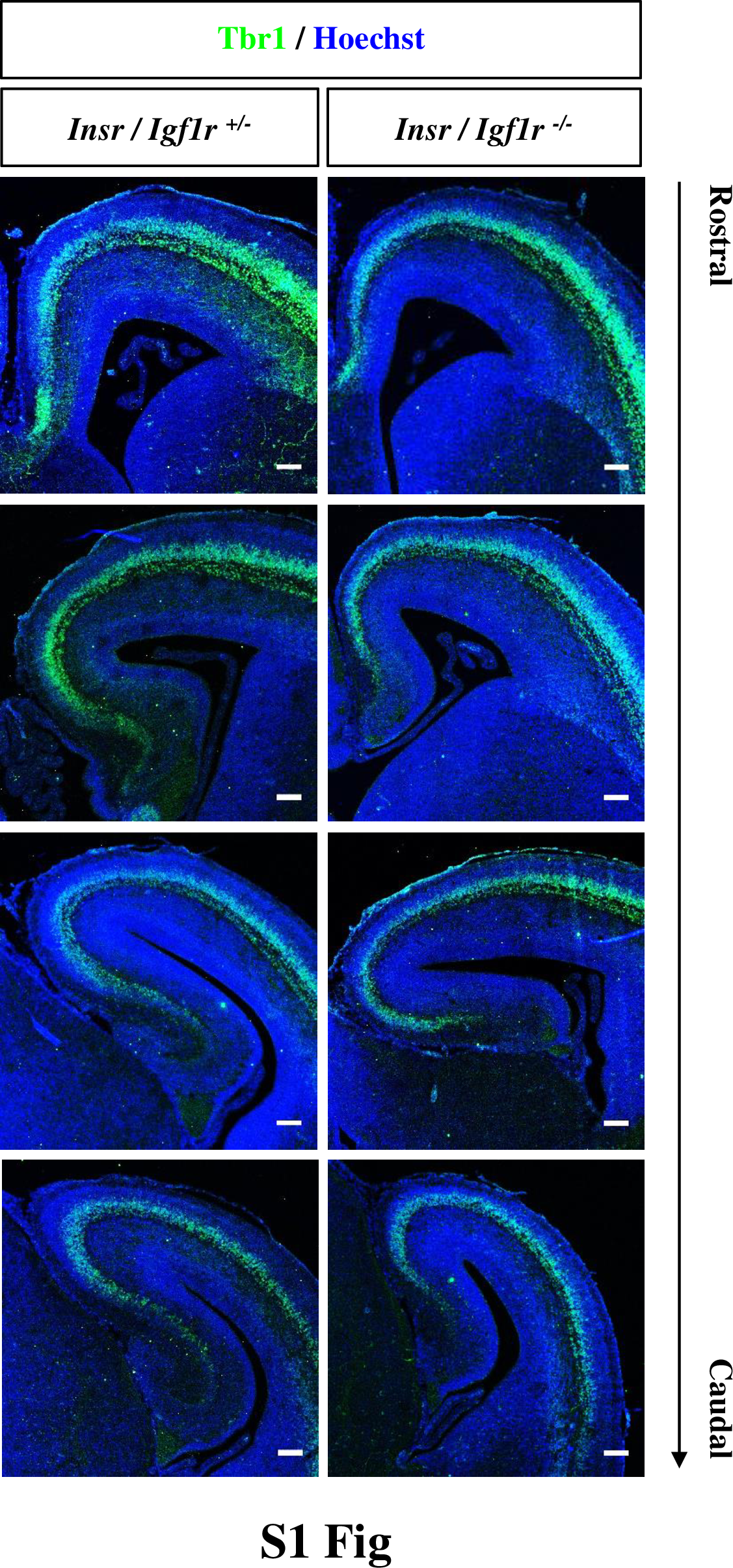

Supplement: S1 Fig — Coronal sections from the cortex of heterozygous (Ifg1r/Insr+/-) and homozygous (Igf1r/Insr-/-) E17 knockout embryos were stained with an anti-Tbr1 antibody (green) and Hoechst 33342 (blue). Sections were selected for analysis every 60 to 80 μm in the rostral to caudal direction beginning with the appearance of the corpus callosum. The scale bar is 100 μm. (TIF) [file pone.0219362.s001.tif]

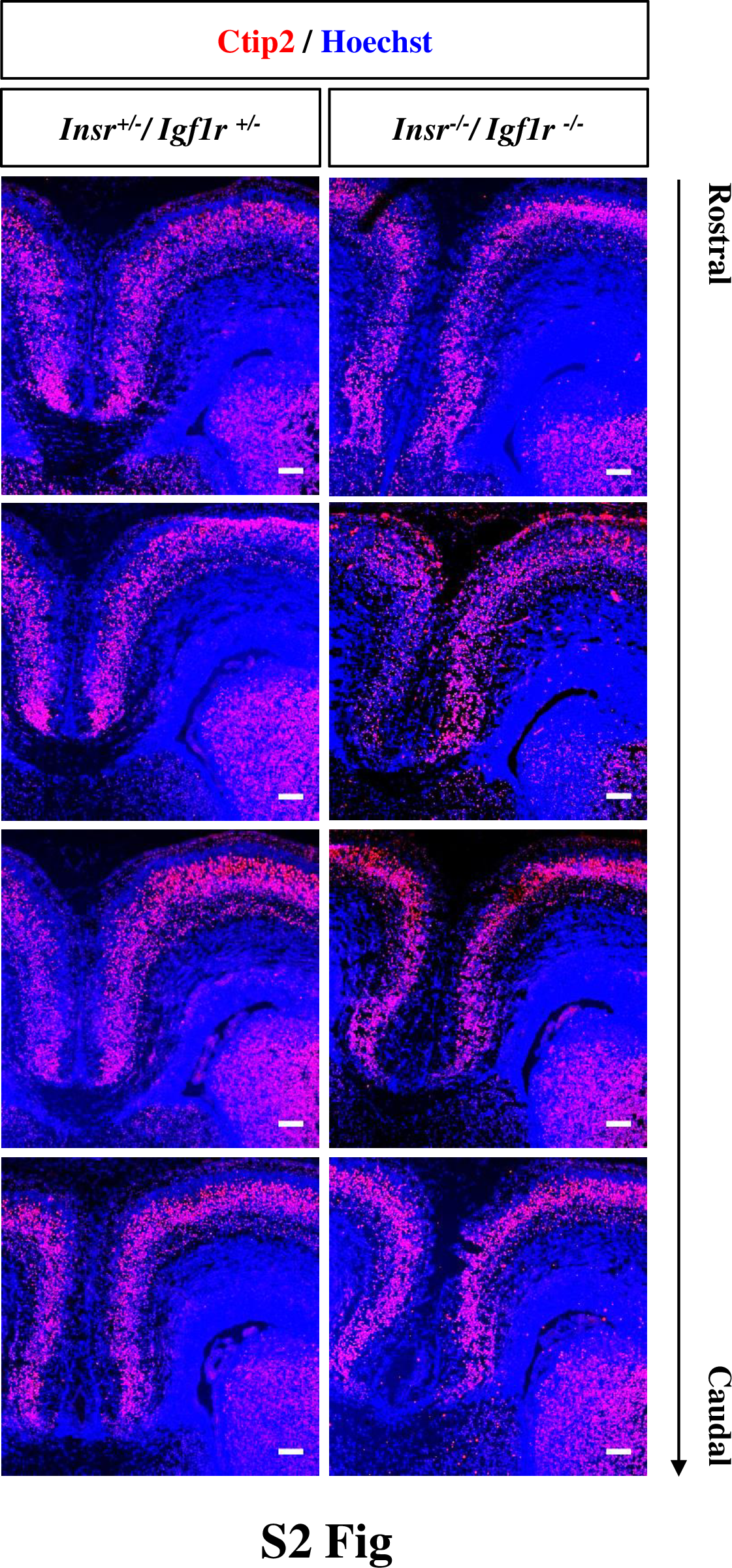

Supplement: S2 Fig — Coronal sections from the cortex of heterozygous (Ifg1r/Insr+/-) and homozygous (Igf1r/Insr-/-) E17 knockout embryos were stained with an anti-Ctip2 antibody (green) and Hoechst 33342 (blue). Sections were selected for analysis every 60 to 80 μm in the rostral to caudal direction beginning with the appearance of the corpus callosum. The scale bar is 100 μm. (TIF) [file pone.0219362.s002.tif]

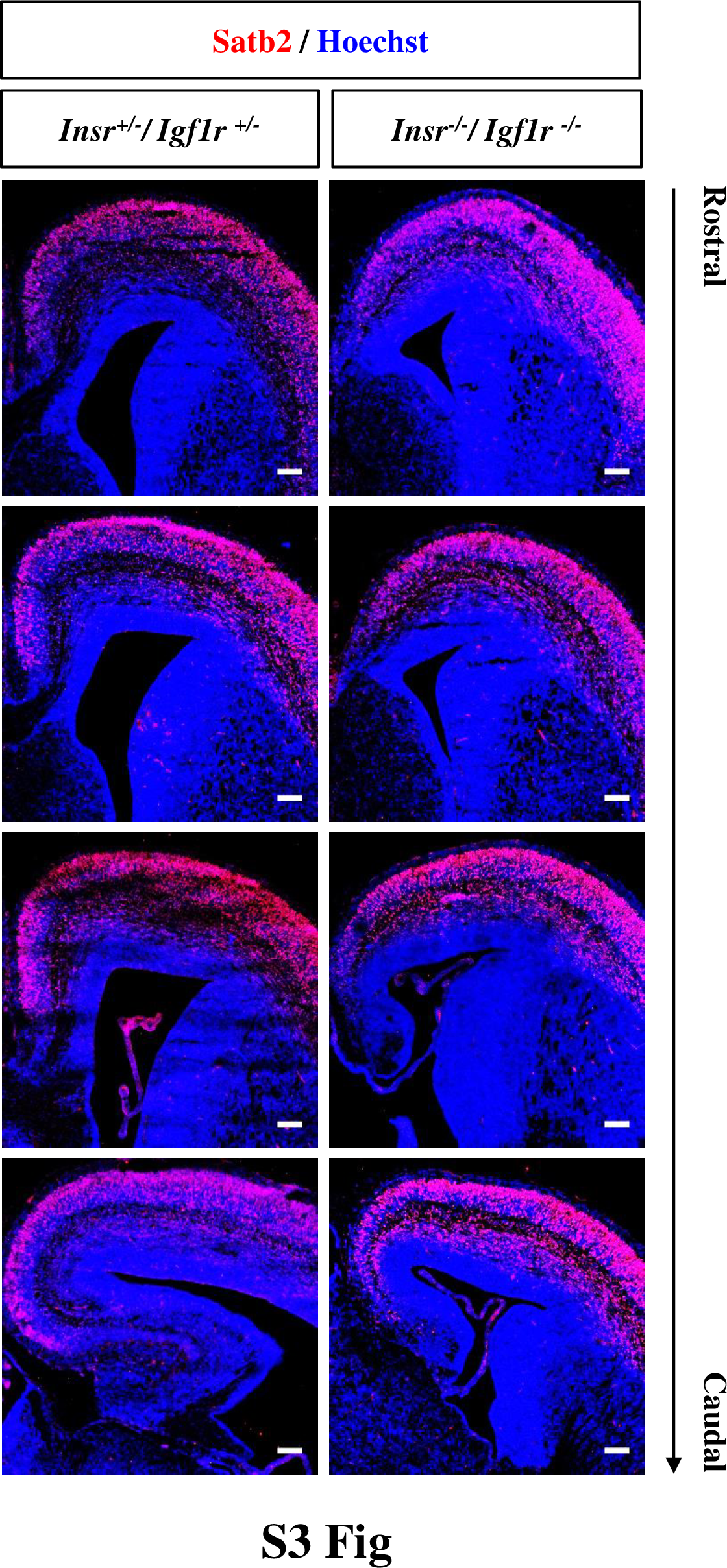

Supplement: S3 Fig — Coronal sections from the cortex of heterozygous (Ifg1r/Insr+/-) and homozygous (Igf1r/Insr-/-) E17 knockout embryos were stained with an anti-Satb2 antibody (green) and Hoechst 33342 (blue). Sections were selected for analysis every 60 to 80 μm in the rostral to caudal direction beginning with the appearance of the corpus callosum. The scale bar is 100 μm. (TIF) [file pone.0219362.s003.tif]

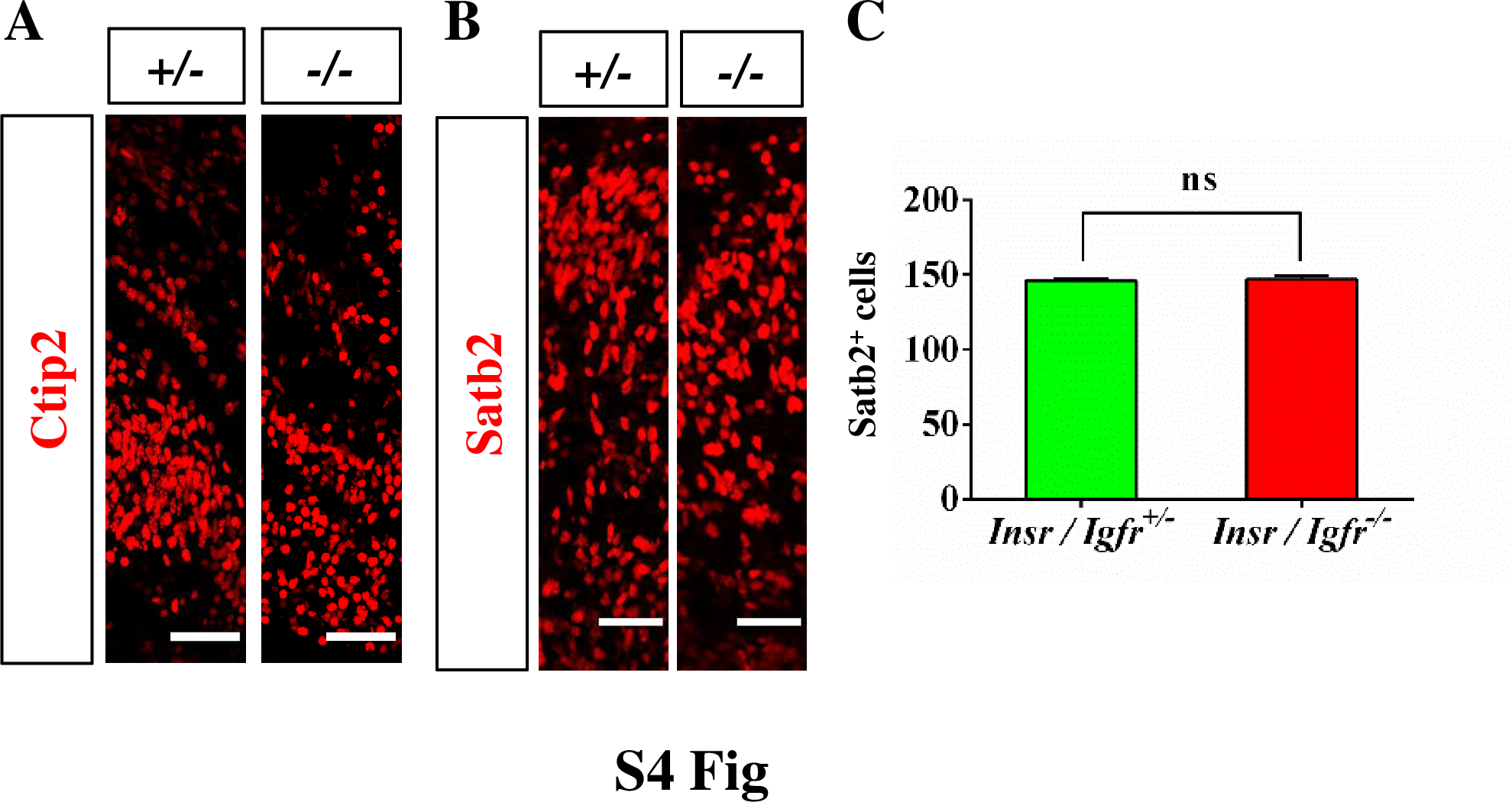

Supplement: S4 Fig — (A, B) Coronal sections from the cortex of heterozygous (+/-: Ifg1r/Insr+/-) and homozygous (-/-: Igf1r/Insr-/-) E17 knockout embryos were stained with an anti-Ctip2 (A, red) or -Satb2 antibody (B, red). The scale bar is 100 μm. (B) The number of cells positive for Satb2 in a column of 100 μm width was quantified in the cingulate cortex of heterozygous and homozygous Igf1r/Insr-Emx1 KO embryos (n = 3 brains; means ± s.e.m.; *, p<0.05; Mann-Whitney U-test). (TIF) [file pone.0219362.s004.tif]

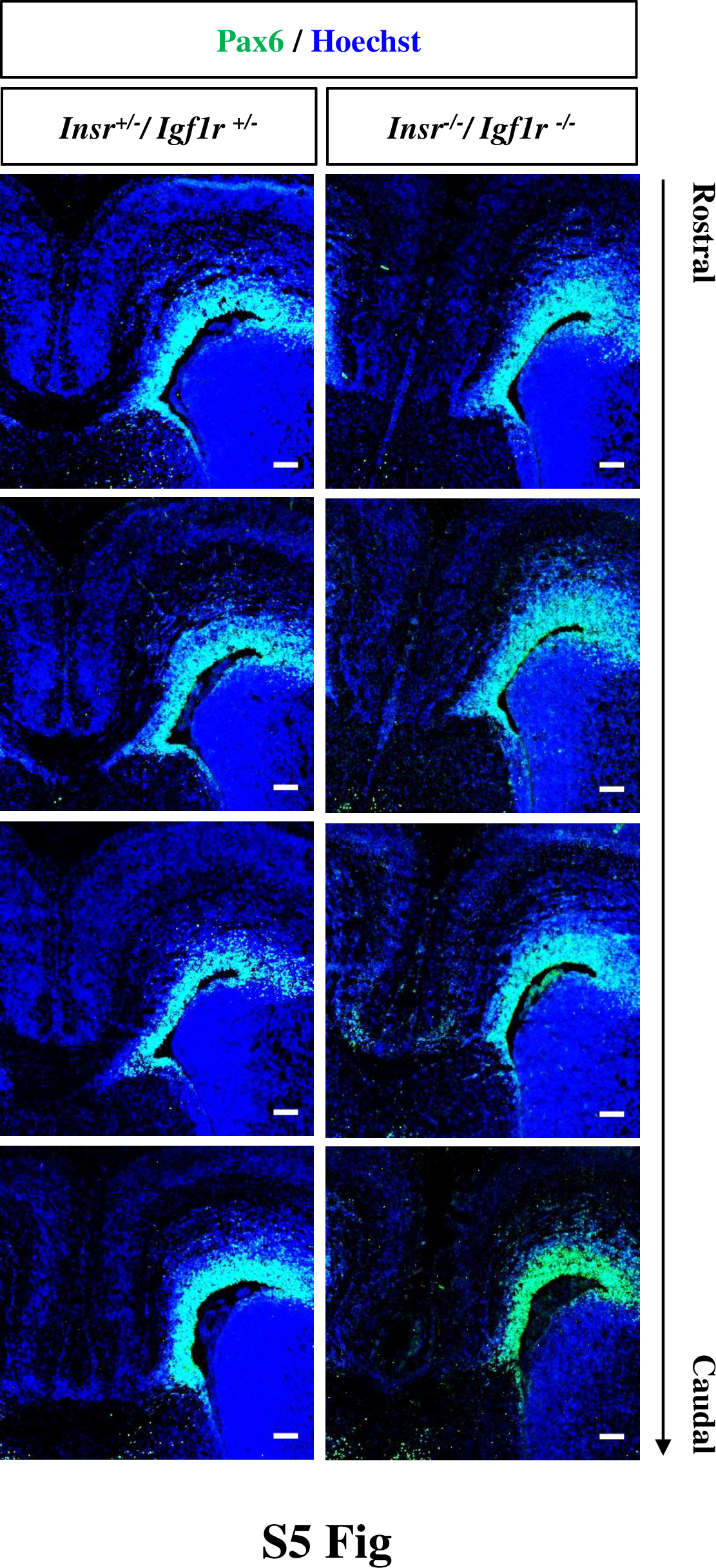

Supplement: S5 Fig — Coronal sections from the cortex of heterozygous (Ifg1r/Insr+/-) and homozygous (Igf1r/Insr-/-) E17 knockout embryos were stained with an anti-Pax6 antibody (green) and Hoechst 33342 (blue). Sections were selected for analysis every 60 to 80 μm in the rostral to caudal direction beginning with the appearance of the corpus callosum. The scale bar is 100 μm. (TIF) [file pone.0219362.s005.tif]

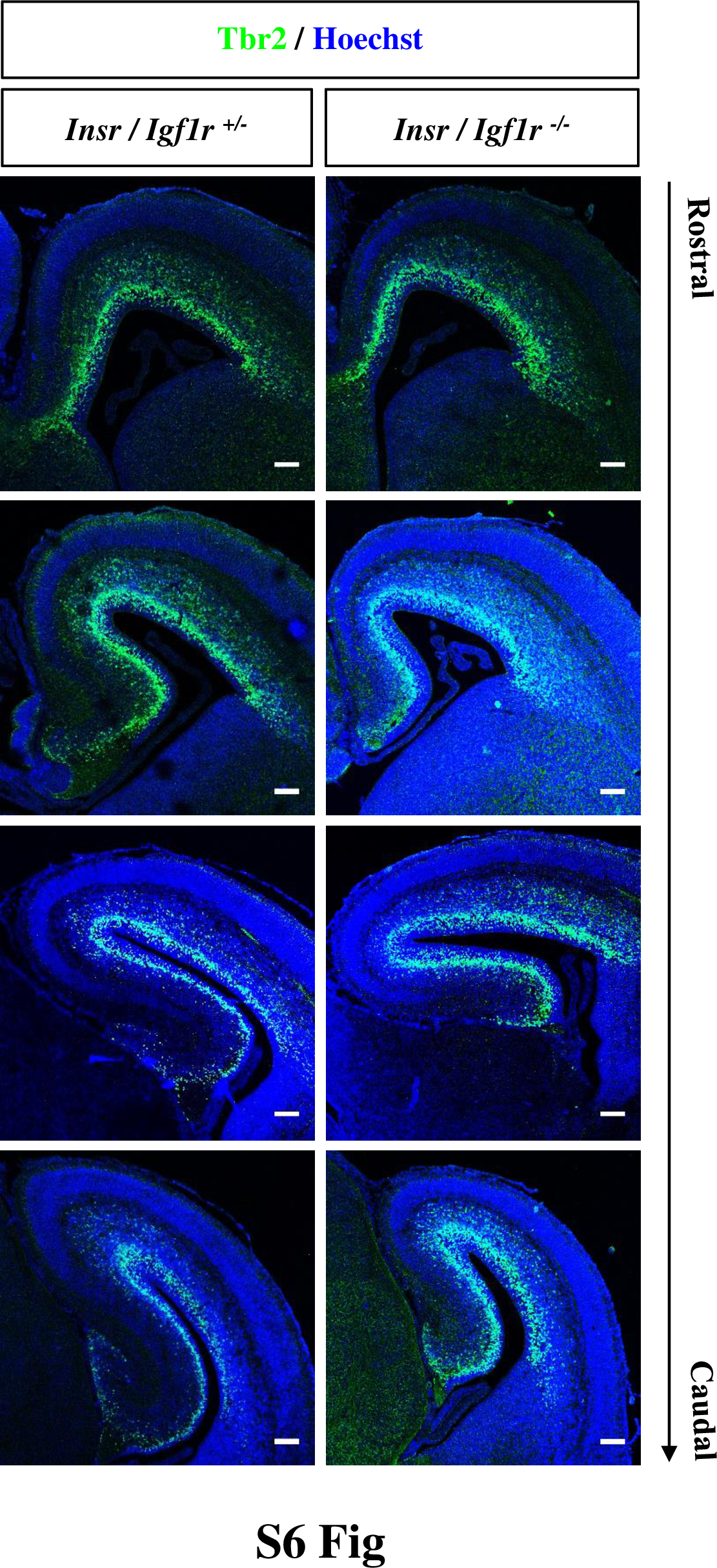

Supplement: S6 Fig — Coronal sections from the cortex of heterozygous (Ifg1r/Insr+/-) and homozygous (Igf1r/Insr-/-) E17 knockout embryos were stained with an anti-Tbr2 antibody (green) and Hoechst 33342 (blue). Sections were selected for analysis every 60 to 80 μm in the rostral to caudal direction beginning with the appearance of the corpus callosum. The scale bar is 100 μm. (TIF) [file pone.0219362.s006.tif]

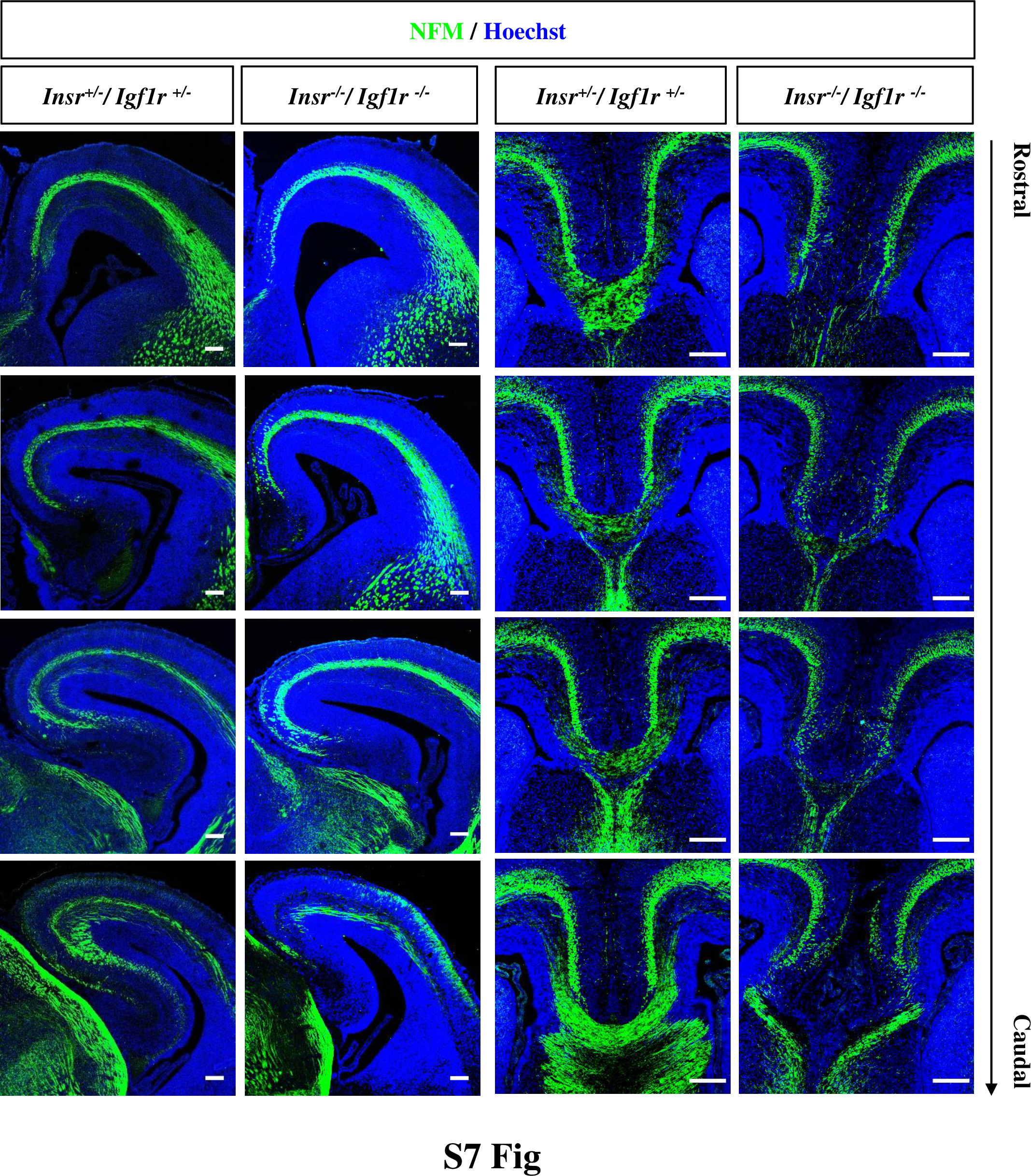

Supplement: S7 Fig — Coronal sections from the cortex of heterozygous (Ifg1r/Insr+/-) and homozygous (Igf1r/Insr-/-) E17 knockout embryos were stained with an anti-NFM (axons, green), antibody and Hoechst 33342 (blue). A higher magnification of the corpus callosum is shown in the right panels. Sections were selected for analysis every 60 to 80 μm in the rostral to caudal direction beginning with the appearance of the corpus callosum The scale bar is 100 μm. (TIF) [file pone.0219362.s007.tif]
